# PGx-Based *in silico* Analyses Identifies the Interactive Role of Genes, Glucose Metabolism and Dopaminergic Dysfunctional Pathways with Chronic Cocaine Use and Misuse

**DOI:** 10.2174/011570159X390146250831143523

**Published:** 2025-09-23

**Authors:** Alireza Sharafshah, Panayotis K. Thanos, Albert Pinhasov, Abdalla Bowirrat, Colin Hanna, Kai-Uwe Lewandrowski, Christopher Rowan, Igor Elman, Mark S. Gold, Catherine A. Dennen, Edward J. Modestino, Rajendra D. Badgaiyan, David Baron, Brian Fuehrlein, Ashim Gupta, Jean Lud Cadet, Aryeh R. Pollack, Jag Khalsa, Milan Makale, Alexander P.L. Lewandrowski, Kenneth Blum

**Affiliations:** 1 Cellular and Molecular Research Center, School of Medicine, Guilan University of Medical Sciences, Rasht, Iran;; 2 Department of Molecular Biology, Adelson School of Medicine, Ariel University, Ariel, Israel;; 3 Behavioral Neuropharmacology and Neuroimaging Laboratory on Addictions, Clinical Research Institute on Addictions, Department of Pharmacology and Toxicology, Jacobs School of Medicine and Biosciences, State University of New York at Buffalo, Buffalo, NY, USA;; 4 Department of Orthopaedics, Fundación Universitaria Sanitas Bogotá, D.C., Bogotá, Colombia;; 5 Center for Advanced Spine Care of Southern Arizona, Tucson, AZ, USA;; 6 Department of Orthopaedics, Universidade Federal do Estado do Rio de Janeiro, Rio de Janeiro, Brazil;; 7 Cambridge Health Alliance, Harvard Medical School, Cambridge, MA., USA;; 8 Department of Psychiatry, Washington University School of Medicine, St. Louis, MO, USA;; 9 Department of Family Medicine, Jefferson Health Northeast, Philadelphia, PA, USA;; 10 Brain & Behavior Laboratory, Department of Psychology, Curry College, Milton, MA, USA;; 11 Division of Clinical Neurology, The Blum Institute of Neurogenetics & Behavior, Austin, TX., USA;; 12 Department of Psychiatry, School of Medicine, Case Western Reserve University, Cleveland, OH., USA;; 13 Division of Addiction Research & Education, Center for Sports, Exercise, and Mental Health, Western University of Health Sciences, Pomona, CA., USA;; 14 Yale University School of Medicine, Yale-New Haven Hospital, New Haven, Connecticut, United States;; 15 Psychiatric Emergency Room, Yale University, VA, Connecticut, USA;; 16 Future Biologics, Lawrenceville, GA, 30043, USA;; 17 Molecular Neuropsychiatry Research Branch, NIH National Institute on Drug Abuse, Baltimore, MD, 20892, USA;; 18 Department of Medicne, School of Medicine, Maryland University, Baltimore, MD., USA;; 19 Department of Radiation Medicine, Oncology, and Applied Sciences, UC San Diego, La Jolla, CA, USA;; 20 Department of Biological Sciences, Dornsife College of Letters, Arts, and Sciences, University of Southern California, Los Angeles, CA., USA

**Keywords:** Pharmacogenomics, cocaine use disorder, glucose metabolism, reward dysregulation, pre-addiction, dopaminergic system, epigenetics, genetics, reward deficiency syndrome (RDS)

## Abstract

**Introduction:**

Our team conducted a pharmacogenomics (PGx) analysis to evaluate the interactions between cocaine, glucose metabolism, and functional connectivity using in-depth silico PGx methods.

**Methods:**

Utilizing PharmGKB, we extracted PGx annotations related to cocaine, glucose, and dopamine (raw data). After filtering, we refined a list of 49 unrepeated, brain-expressed genes and examined their interactions in a protein-protein interaction (PPI) network through STRING-MODEL, identifying top candidate genes.

**Results:**

Targeting key protein-coding genes with the highest connectivity, we identified *COMT*, *DRD2*, and *SLC6A3*, along with their 17 connected genes. A deep dive into gene-miRNA interactions (GMIs) using NetworkAnalyst revealed that *COMT*, *DRD2, *and hsa-miR-16-5p have multiple interactions with *OPRM1 *and *BDNF*. Enrichment analysis *via* Enrichr confirmed that this refined set of 17 impacts dopamine function and are interactive with dopaminergic pathways. Notably, Substance Use disorders (SUD) were the most significant manifestation predicted for the interplays among these genes.

**Discussion:**

Reviewing all PGx annotations for the 17 genes, we found 4,665 PGx entries, among which 1,970 were significant, with a *p*-value above 0.045. These were ultimately filtered down to 32 potential PGx annotations excluded in association with “Cocaine,” “Glucose or Diabetes,” and “Dopamine”. Accordingly, 12 Pharmacogenes represented 32 PGx-associated with Cocaine, Glucose, and Dopamine, including *DRD2, COMT, OPRD1, OPRM1, SLC6A3, CHRNA5, CNR1, CYP2C19, DBH, GABRA2, NOS1AP,* and *SYT1.*

**Conclusion:**

This *in silico* PGx analysis demonstrates strong, validated connections based on prior published data and robust computational predictions. Among the findings, the *COMT * gene was found to be the best-scoring gene here.

## 1 INTRODUCTION

Cocaine use and associated hospitalizations have surged in recent years, paralleling the increase in Cocaine Use Disorder (CUD). Cocaine’s detrimental effects on mental and physical health—both in the short term and long term—are well-documented. Established effects include elevated blood pressure, heightened risk of heart disease, and severe damage to the respiratory and nervous systems [1-14]. Cocaine’s impact on the brain is extensive, causing damage to veins and arteries that can lead to chronic headaches, stroke, seizures, and seizure disorders [15-23].

Current literature supports cocaine’s inhibitory effect on brain glucose utilization in humans [24]. Dysfunction is primarily localized within the cortico-striatal-thalamic circuitry, which plays a key role in the reward systems that sustain drug use and cravings. Chronic cocaine use, commonly linked with CUD, is associated with a range of cognitive deficits such as reduced attention, impaired memory, decreased verbal fluency, sensory-perceptual challenges, weakened response inhibition, and increased impulsivity [25-28]. These cognitive issues are consistent with observed disruptions in brain glucose metabolism.

Preclinical research has demonstrated that cocaine induces region-specific alterations in cerebral blood flow and glucose utilization after a single dose, impacting task-related performance [29]. In rodent models, cocaine enhances dopaminergic signaling and motor function by blocking dopaminergic reuptake *via* dopamine transporter (DAT) inhibition [30, 31]. It also induces adaptations in cortical regions associated with inhibitory avoidance, particularly in cortical pyramidal neurons [32-34]. Fluorodeoxyglucose positron emission tomography (FDG-PET) is widely employed in animal studies to measure the brain’s glucose response to drugs and therapeutic approaches [35-43]. Cocaine Self-Administration (CSA) is a standard approach for examining cocaine abuse-related behavior in rodent models [44-46]. Animal studies have found alterations in brain activity following CSA, aligning with findings on glucose utilization in humans with cocaine dependence [47-49]. There is strong evidence linking cocaine use, particularly CSA, to dopamine-mediated reward circuits [50-53], with cocaine abuse disrupting both cortical and sub-cortical functions, leading to withdrawal and preoccupation [54, 55].

Repeated cocaine exposure leads to altered histone acetylation and methylation on lysine residues and DNA within the nucleus accumbens (NAc). Notably, Nestler’s group investigated histone arginine methylation in reward-processing models, whereas Damez-Werno *et al*. [56] observed decreased *PRMT6* and *H3R2me2a* levels in the NAc of rodents and cocaine-dependent humans engaged in self-administration experiments. *PRMT6* overexpression in *dopamine receptor type 2-expressing medium spiny neurons* (*D2-MSNs)* increased cocaine-seeking behaviors, while *PRMT6* overexpression in *dopamine receptor type 1-expressing medium spiny neurons* (*D1-MSNs)* provided a protective effect. This finding suggests that dopaminylation (*H3R2me2a* binding) may occur in psychostimulant use disorder (PSU), where binding inhibitor Srcin1—similar to the *DRD2 A2* allele polymorphism—protects against psychostimulant-seeking behavior by normalizing NAc dopamine expression. Multiple studies corroborate the association between the *DRD2 Taq A1* allele and severe cocaine dependence. Lepack *et al*. (2020) found that acute cocaine exposure increases dopamine levels in NAc synapses, leading to histone H3 glutamine 5 dopaminylation (*H3Q5dop*) and inhibition of *D2* expression. Chronic exposure heightens this inhibition, which is linked to withdrawal symptoms. During cocaine withdrawal, Src kinase signaling inhibitor 1 (*Srcin1* or *p140CAP*) reduces *H3R2me2a* binding, inducing a “homeostatic brake.” Decreased Src signaling within NAc *D2-MSNs* restores dopamine balance in the NAc, reducing cocaine-induced reward effects and, therefore, motivation for self-administration [57-60].

Recent studies emphasize the importance of resting-state functional connectivity in brain reward pathways, especially in cases of chronic cocaine abuse or dependence [61]. High-risk individuals often exhibit a reduced response to stimuli, potentially as a result of DNA polymorphisms or epigenetic changes associated with cocaine use [62]. This may lead to a diminished ‘thrill,’ possibly due to genetic predispositions for addiction [63]. We suggest utilizing a validated genetic assessment tool (such as GARS) in conjunction with pro-dopamine regulation (KB220) as a frontline modality for cocaine dependence and opioid use disorder (OUD), reserving potent opioids for short-term harm reduction when necessary [64].

Gene editing holds promise for addressing adverse genetic modifications early in life, which may be influenced by environmental factors and passed down across generations, leading to neurotransmitter imbalances typical of Reward Deficiency Syndrome (RDS). Through global scientific exploration, we aspire to create a future where individuals are liberated from chronic pain and illness, attaining an optimal state of well-being akin to an optimal state of homeostasis [65].

Our team evaluated the interaction of cocaine, glucose metabolism, and functional connectivity using deep silico PGx analyses. This work utilized PharmGKB to extract PGx annotations related to cocaine, glucose, and dopamine. Fig. (**1**) provides a high-level schematic of our PGx analyses.

## 2 MATERIALS AND METHODS

To obtain the raw data for Cocaine, Glucose, and Dopamine, we started with the PharmGKB database (https://www.pharmgkb.org/) and searched these keywords separately. Then, each dataset was filtered for significant Variant Annotations based on *p*-value <0.05, which was performed separately for each individual dataset (cocaine, glucose, dopamine) prior to merging. Subsequently, all datasets were combined together, and duplications were removed. Moreover, we checked every single gene for having expression in the brain by GeneCards (http://www.genecards.org/), and the rationale behind that was particularly the biological relevance of that gene to cocaine's neurological mechanisms. Importantly, Protein-Protein Interactions (PPIs) were conducted on all the refined 17 genes by STRING-MODEL (https://string-db.org/), and unconnected genes were discarded. Following the selection of the highly impacted protein-coding genes, they were thus considered for further analyses to validate their relationship to cocaine abuse, alterations linked to glucose metabolism, and functional connectivity. To accomplish this, we carried out Gene-miRNA Interactions (GMIs) by NetworkAnalyst (https://www.networkanalyst.ca/ NetworkAnalyst/) and lastly, we employed Enrichr (https://maayanlab.cloud/Enrichr/) for the multi-filtered highly potential genes in various categories, mainly Pathway Analysis based on KEGG and Reactome, Gene ontologies, and Diseases/Drugs Assessments. All the details and references for the basic structure of the current strategy of study design and analyses are summarized in Table **1**. Additionally, our recent paper can facilitate understanding of the PGx-based strategy of our team [66].

Remarkably, a flowchart visualized in Fig. (**1**) summarizes all steps of our strategy pipeline with a short description for each step. This figure highlights the input, prioritized, and output data for the current in-depth silico PGx and systems biology investigations.

## 3 RESULTS

To explore known interactions of PGx annotations related to cocaine, glucose metabolism, and dopamine, we accessed multiple large databases. Our approach involved a structured *in silico* analysis, beginning with the initial question of how genes and phenotypic pathways—such as dopamine dysregulation—interact with PGx annotation data. We refined the raw data through multiple filtering steps, followed by primary and deep *in silico* assessments. Interestingly, we identified PGx annotations that included nine dopamine-related genes, with *DRD2* and *SLC6A3* overlapping in a cocaine PGx-derived gene group. Moreover, we also found that *COMT* was common in the glucose metabolism group. Subsequently, our analysis focused on hub genes, specifically highlighting *COMT* (the primary linking gene with 13 PPIs), *DRD2* (with 10 PPIs), and *SLC6A3* (with 8 PPIs). STRING-MODEL analysis revealed that *BDNF, OPRM1*, and *DBH* connect with all three hub genes (*COMT, DRD2*, and *SLC6A3*), as shown by the blue arrows in Fig. (**2**). *COMT* appears to have a joint role across all three categories (cocaine, glucose metabolism, and dopamine agents). Our multi-database PGx analyses show that *COMT*'s function is influenced by epigenetic mechanisms impacting all three phenotypes, potentially offering further therapeutic clues.

Furthermore, we focused on the hub genes in this list (*COMT, DRD2,* and *SLC6A3*) and their direct connections. Specifically, 17 unique genes remained including *COMT, DRD2, SLC6A3, BDNF, DRD3, OPRM1, OPRD1, DBH, ABCB1, CHRNA5, CNR1, CYP2C19,* *ESR1, GABRA2, NOS1AP, SYT1,* and *UGT2B15*. We performed another STRING-MODEL to further validate the PPIs of these genes with each other (Fig. **3**).

Additionally, we employed NetworkAnalyst to investigate the Gene-miRNA Interactions (GMIs) and found hsa-miR-16-5p has connections with *COMT*, *OPRM1,* and *BDNF*. Notably, *BDNF* is linked with *ESR1* by two miRNAs, including *hsa-miR-22-3p* and *hsa-miR-206*. Moreover, we found that *ESR1* is connected to *DRD2* with *hsa-miR-9-5p*. We can conclude that *COMT* and *DRD2* are involved in the main GMIs that were derived from resulted from the 17 potential genes from PPIs (Fig. **4**).

In order to further understand the interactiveness of the 17 refined genes, we performed an Enrichment analysis by using Enrichr tool. These results classified distinct categories of these genes that included Pathway analysis, Gene Ontologies (GO), Drug/Disease Analysis, and Metabolomics predictions are presented herein.

### 3.1 Pathway Analysis

Pathway analysis of the candidate genes according to the KEGG database revealed that neuroactive ligand-receptor interaction was found to be the most plausible pathway (q-value=3.18E-07; OR=41.18). Remarkably, as the second most significant pathway, the Dopaminergic synapse had the highest scores (q-value=9.69E-05; OR= 47.73) compared with Cocaine addiction (q-value=1.47E-04). These results reflect the predominance of the dopaminergic system on cocaine and other pathways listed in Table **2**.

### 3.2 GO Assessments

Gene ontology assessments were performed involving three subdivisions, including GO Biological process, GO Cellular component, and GO molecular function. The results indicated the following: Dopamine Metabolic Process (GO:0042417) (q-value=4.26E-07; OR=409.6) in GO Biological process, Neuron Projection (GO:0043005) (q-value=2.88E-07; OR=31.47) in GO cellular component, and G Protein-Coupled Receptor Activity (GO:0004930) (q-value=1.3E-04; OR=33.57) processes (Table **3**).

### 3.3 Diseases/Drugs Assessments

In a Diseases/Drug Assessment according to DisGeNET and GeDiPNet databases, Substance abuse problem (q-value=1.98E-19; OR=332.98) represented the most significant plausible manifestation resulting from the interplay among the candidate genes (Table **4**).

### 3.4 Metabolomics Workbench Metabolites

Obviously, from Table **5**, the results of metabolomics indicate that dopamine, with a q-value of 3.26E-04, is the most significant metabolite interacting with the candidate genes.

### 3.5 PGx of Hub Genes and their Direct Connections

To get a better view in Personalized Medicine options following suggesting the hub genes and their connected genes, we checked all PGx annotations of 17 genes (Fig. **2**) and found 4,665 PGx annotations related to the aforementioned genes; among them, 1,970 significant PGx annotations with a p-value higher than 0.045 filtered and finally, 32 potential PGx annotations excluded in association with “Cocaine”, “Glucose or Diabetes”, and “Dopamine”. Accordingly, 12 Pharmacogenes represented 32 PGx-associated with Cocaine, Glucose, and Dopamine, including *DRD2, COMT, OPRD1, OPRM1, SLC6A3, CHRNA5, CNR1, CYP2C19, DBH, GABRA2, NOS1AP,* and *SYT1* (Table **6**)*.*

## 4 DISCUSSION

There is a critical need for FDA-approved medications to treat Psychostimulant Use Disorder (PUD). The neurophysiological impact of these highly addictive substances leads to both acute and chronic neurological deficits, which can result in severe cardiovascular issues, including the risk of sudden stroke.

This article presents an in-depth silico PGx analysis related to cocaine abuse, examining its effects on glucose metabolism [73] and the genes and pathways linked to psychiatric and neurobiological sequelae of CUD, specific neurogenetic associations are influenced by epigenetic factors. The authors suggest that mild stimulation of *DRD2* receptors through specific treatment modalities can induce dopamine release, in turn modulating *DRD2*-targeted mRNA and improving *DRD2* receptor function in humans. This proliferation of *DRD2* receptors, in turn, will attenuate craving behavior, particularly in genetically vulnerable high-risk populations [74]. Understanding the molecular neurogenetic connections between opioids, mesolimbic dopamine, and "wanting" supports this hypothesis [75-77].

As both scientific and clinical communities seek FDA-approved treatments for PUD, the induction of dopamine homeostasis through activation of brain reward pathways presents a promising approach. This strategy offers treatment for neurotransmitter functional deficits, potential prophylaxis, and supports recovery processes. In particular, dopamine regulation may help individuals overcome the effects of a hypodopaminergic ditch.

Our team conducted an in-depth silico PGx analysis to examine the interactions between cocaine, glucose metabolism, and functional connectivity. Utilizing data from PharmGKB, we extracted PGx annotations related to cocaine, glucose, and dopamine. Through rigorous filtering, we refined a list of 49 unrepeated, brain-expressed genes and analyzed their interactions within a PPI network *via* the STRING-MODEL, ultimately identifying top candidate genes. Specifically, we focused on the most interconnected protein-coding genes—*COMT*, *DRD2*, and *SLC6A3*—along with their 17 connected genes. Enrichment analysis using Enrichr revealed that 17 genes influence dopamine function and interact with dopaminergic pathways. Substance Use Disorder (SUD) emerged as the most significant manifestation predicted among these genes.

While the role of *COMT* and cocaine abuse is well known [78-85], there is also convincing evidence for both *DRD2* [86-95] and *SLC6A3 (DAT1)* [96-105] genes, with risk alleles in these genes being associated with increased vulnerability to cocaine and even crack dependence. Obviously, there were some limitations which this paper faced with them and we highly recommend them to be addressed and considered in the future studies; for example, the entry data obtained from GWAS catalog lacks some demographic information such as sex specification (male/female) for each GWAS or in total; there are well-known genes (*e.g*. *ESR1*) which have different genetic algorithms (regulation and expression patterns) in males *vs.* females vise vera; also, the other recently published GWA studies may not be included in this study which should not be neglected in the future investigations.

## CONCLUSION

We are proposing herein that these PGx-based *in silico* analyses provide reliable, strong, validated connections based on the prior published data and highly accurate computational predictions. Notably, the *COMT* gene in our study was found to be the best-scoring gene in our analyses. These results further support the importance of the genetic basis of reward deficiency, strongly linked to dopaminergic dysregulation. We are encouraging more research focused on novel neurobiological interventions, including ways to target reward dopaminergic genetic DNA antecedents as a pre-addiction type phenotype. Additionally, investigations on the 12 Pharmacogenes with introduced 32 PGx annotations are highly recommended for further assessments in future studies.

## Figures and Tables

**Fig. (1):**
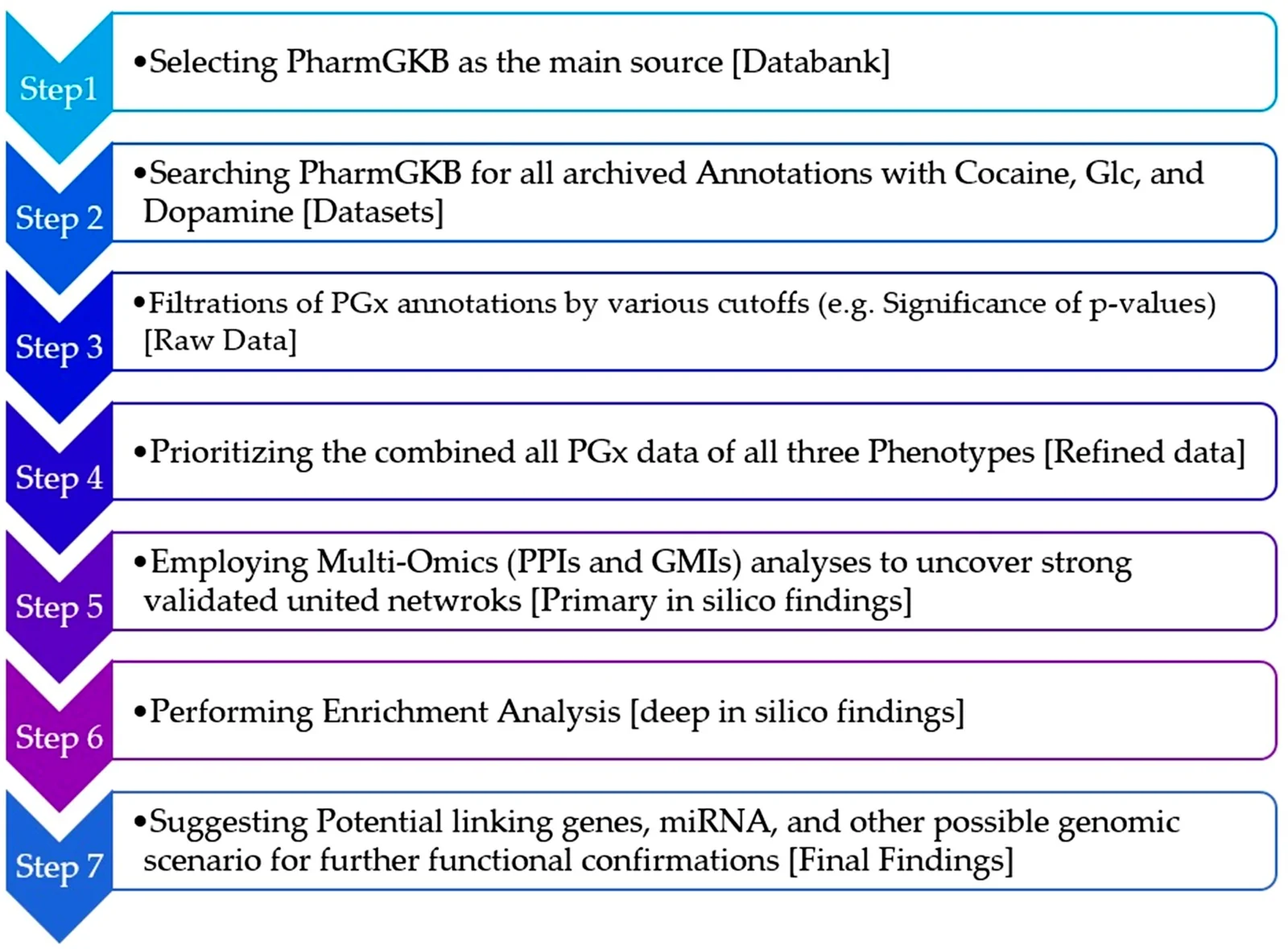
**Fig. (1).** Flowchart scheme of strategy utilized in this study, from Databank selection to Final findings through 7 specific steps. **Abbreviation**: Glc: Glucose; PGx: Pharmacogenomics; PPIs: Protein-Protein Interactions; and GMIs: Gene-miRNA Interactions.

**Fig. (2):**
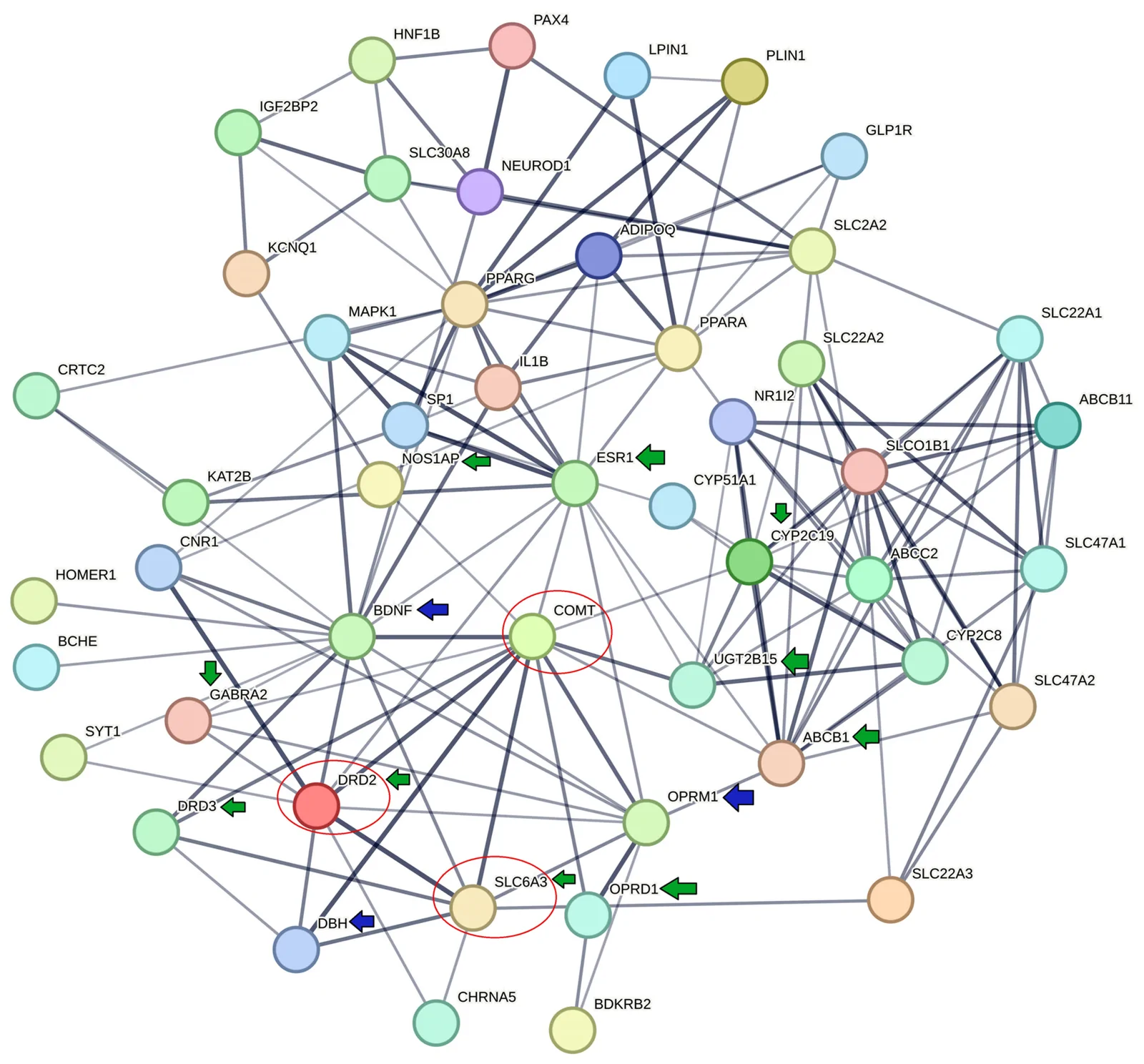
STRING-MODEL resulted from the combination of PGx annotations (obtained from PharmGKB), including three main categories: Cocaine, Glucose Metabolism, and Dopamine agents. *COMT* is the most promising gene which is common among the three categories with 13 connections, followed by *DRD2* (10 connections), and *SLC6A3* (8 connections). Green arrows refer to *COMT* connections, and blue arrows show the common protein-coding genes linked to all three top-scored genes (*COMT*, *DRD2*, *SLC6A3*).

**Fig. (3):**
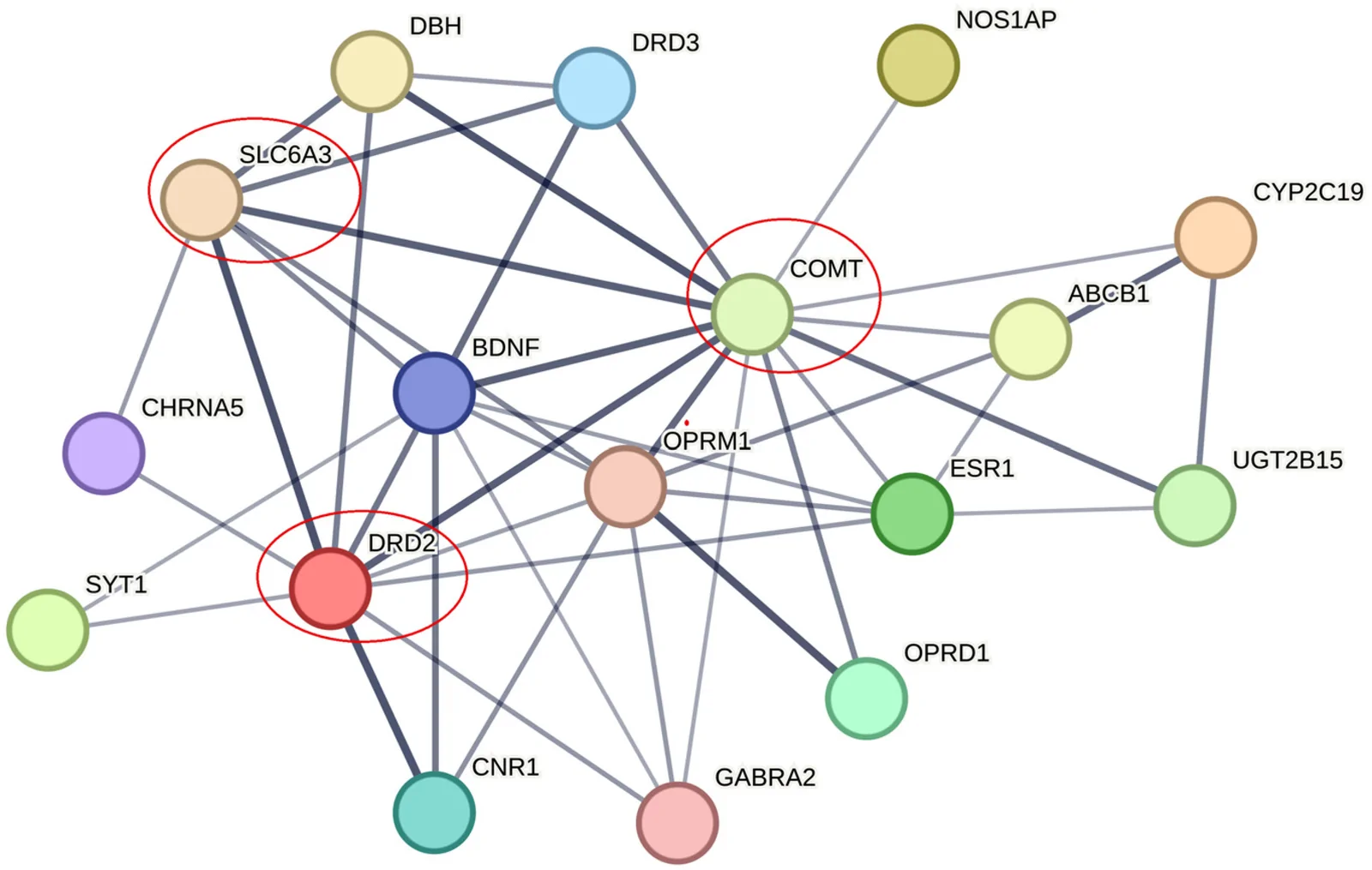
STRING-MODEL of potential hub genes (*COMT*, *DRD2*, and *SLC6A3*) with their direct connections (red circles).

**Fig. (4):**
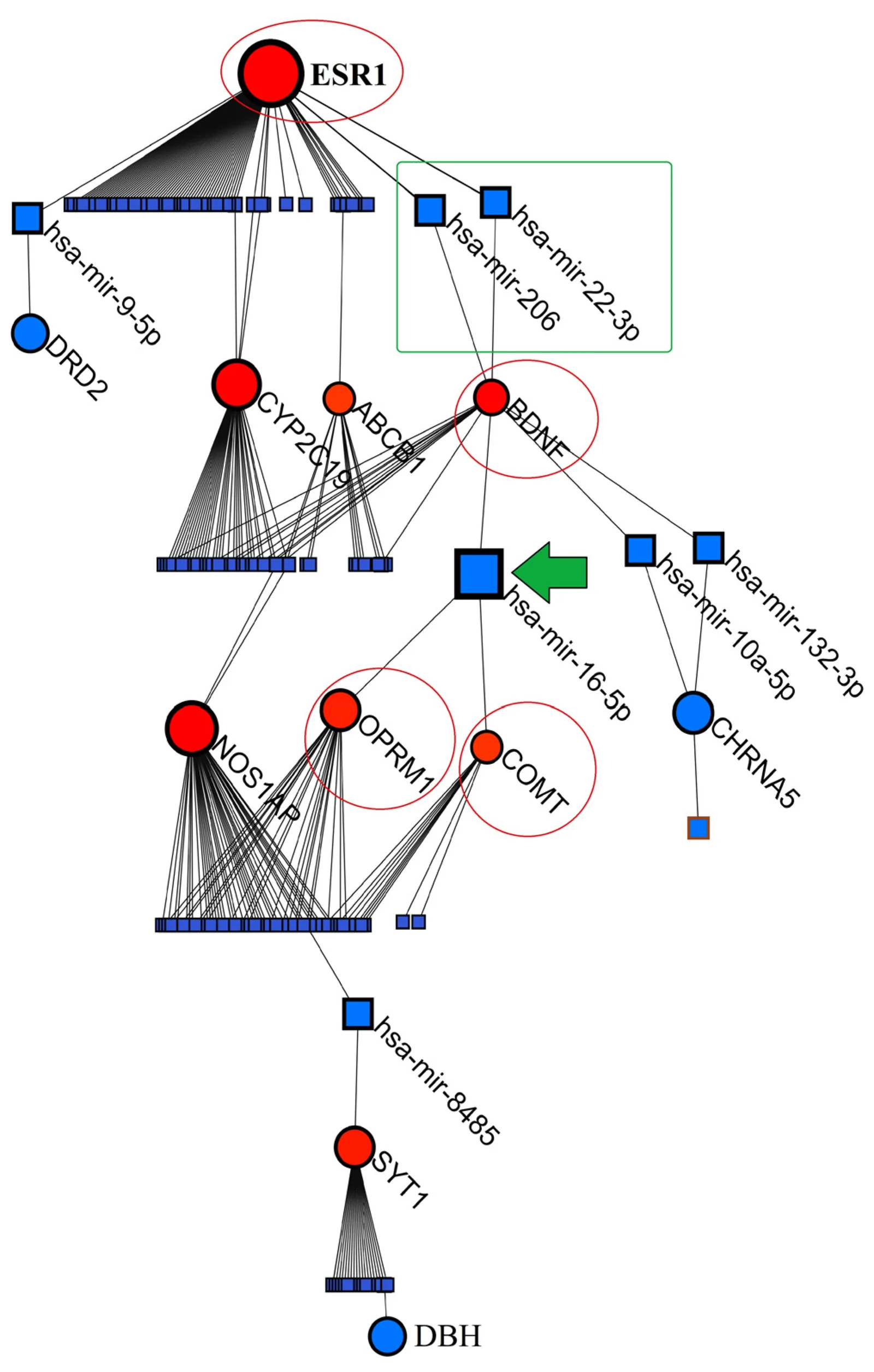
Gene-miRNA Interactions (GMIs) of potential genes from PPI assessments in a Sugiyama model. hsa-miR-16-5p represents the most important miRNA compared with the other miRNAs, which link *COMT* and *OPRM1* to *BDNF*. Notably, *DRD2* is connected to *ESR1* *via* hsa-miR-19-5p.

**Table 1:** Details and references of all in silico databases and systems biology tactics applied in this paper.

**Level**	**Database**	**Site**	**Software (Version)**	**References [Doi]**
PGx	VAA	https://www.pharmgkb.org/	PharmGKB	[67]
PPIs	STRING-MODEL	https://string-db.org/	STRING (12.0)	[68]
GRNs	GMIs (miRTarBase)	https://mirtarbase.cuhk.edu.cn /~miRTarBase/miRTarBase_2022/php/index.php	NetworkAnalyst (3.0)	[69]
EA	Pathway Analysis	https://maayanlab.cloud/Enrichr/	Enrichr	[70]
	GO	https://maayanlab.cloud/Enrichr/	Enrichr	[71]
	DDA	https://maayanlab.cloud/Enrichr/	Enrichr	[72]

**Abbreviations**: PGx: Pharmacogenomics; VAA: Variant Annotation Assessment; PPIs: Protein-Protein Interactions; GRNs: Gene Regulatory Networks; GMIs: Gene-miRNA Interactions; EA: Enrichment Analysis; GO: Gene Ontology; DDA: Diseases Drugs Assessment.

**Table 2:** Pathway analysis outputs based on KEGG and Reactome databases for 17 candidate genes.

**Index**	**Name**	***p*-value**	**q-value**	**OR**
KEGG	Neuroactive ligand-receptor interaction	6.62E-09	3.18E-07	41.18
KEGG	Dopaminergic synapse	4.04E-06	9.687E-05	47.73
KEGG	Cocaine addiction	9.17E-06	1.468E-04	92.87
Reactome	Transmission Across Chemical Synapses R-HSA-112315	1.48E-06	1.571E-04	34.13
Reactome	Dopamine Receptors R-HSA-390651	6.79E-06	3.599E-04	888
Reactome	Neuronal System R-HSA-112316	1.33E-05	4.712E-04	21.44
Reactome	Neurotransmitter Clearance R-HSA-112311	3.05E-05	8.077E-04	332.92
Reactome	Drug ADME R-HSA-9748784	6.73E-05	1.426E-03	46.33
Reactome	Class A/1 (Rhodopsin-like Receptors) R-HSA-373076	1.41E-04	2.493E-03	18.73
KEGG	Tyrosine metabolism	4.21E-04	4.693E-03	78.23
KEGG	Alcoholism	4.89E-04	4.693E-03	23.19
Reactome	GPCR Ligand Binding R-HSA-500792	5.1E-04	6.896E-03	13.24
Reactome	Amine Ligand-Binding Receptors R-HSA-375280	5.2E-04	6.896E-03	69.98
Reactome	Aspirin ADME R-HSA-9749641	6.3E-04	7.419E-03	63.3
KEGG	Chemical carcinogenesis	1.012E-03	8.099E-03	17.93
Reactome	Biological Oxidations R-HSA-211859	7.76E-04	8.224E-03	19.7
KEGG	Steroid hormone biosynthesis	1.208E-03	8.285E-03	45.03
KEGG	Synaptic vesicle cycle	1.966E-03	1.18E-02	34.92
KEGG	Bile secretion	2.606E-03	1.279E-02	30.14
KEGG	Morphine addiction	2.664E-03	1.279E-02	29.8

**Note**: q-value and OR refer to adjusted *p*-value and odds ratio.

**Table 3:** Gene ontology assessments of candidate genes classified in three major cellular and molecular processes in the cell.

**Index**	**Name**	***p*-value**	**q-value**	**OR**
GO Cellular Component	Neuron Projection (GO:0043005)	6.71E-09	2.88E-07	31.47
GO Biological Process	Dopamine Metabolic Process (GO:0042417)	1.37E-09	4.26E-07	409.6
GO Biological Process	Phospholipase C-activating G Protein-Coupled Receptor Signaling Pathway (GO:0007200)	6.48E-09	1.01E-06	106.33
GO Cellular Component	Axon (GO:0030424)	6.03E-07	1.3E-05	41.21
GO Biological Process	Catecholamine Metabolic Process (GO:0006584)	1.45E-07	1.5E-05	427.99
GO Biological Process	Response To Ethanol (GO:0045471)	4.13E-07	3.2E-05	285.26
GO Molecular Function	G Protein-Coupled Receptor Activity (GO:0004930)	1.61E-06	1.3E-04	33.57
GO Biological Process	Chemical Synaptic Transmission (GO:0007268)	2.47E-06	1.53E-04	30.65
GO Biological Process	Negative Regulation Of Protein Secretion (GO:0050709)	4.57E-06	2.36E-04	118.73
GO Molecular Function	G Protein-Coupled Opioid Receptor Activity (GO:0004985)	6.79E-06	2.75E-04	888
GO Biological Process	Prepulse Inhibition (GO:0060134)	6.79E-06	2.84E-04	888
GO Biological Process	G Protein-Coupled Receptor Signaling Pathway, Coupled To Cyclic Nucleotide Second Messenger (GO:0007187)	9.75E-06	2.84E-04	90.89
GO Biological Process	Response To Alkaloid (GO:0043279)	1.02E-05	2.84E-04	665.97
GO Biological Process	Response To Cocaine (GO:0042220)	1.02E-05	2.84E-04	665.97
GO Cellular Component	Dendrite (GO:0030425)	6.73E-05	9.65E-04	22.81
GO Molecular Function	Ligand-Gated Monoatomic Ion Channel Activity (GO:0015276)	1.7 E-04	4.597E-03	126.74
GO Molecular Function	Neuropeptide Binding (GO:0042923)	2.54E-04	5.138E-03	102.34
GO Molecular Function	Transmitter-Gated Monoatomic Ion Channel Activity Involved In Regulation Of Postsynaptic Membrane Potential (GO:1904315)	3.75E-04	6.082E-03	83.13
GO Cellular Component	Exocytic Vesicle Membrane (GO:0099501)	9.14E-04	8.154E-03	52.11
GO Cellular Component	Synaptic Vesicle Membrane (GO:0030672)	9.48E-04	8.154E-03	51.11
GO Cellular Component	Caveola (GO:0005901)	1.248E-03	8.943E-03	44.27
GO Cellular Component	Glutamatergic Synapse (GO:0098978)	1.966E-03	1.208E-02	34.92
GO Cellular Component	Flotillin Complex (GO:0016600)	5.09E-03	2.552E-02	249.72
GO Cellular Component	Clathrin-Sculpted Gamma-Aminobutyric Acid Transport Vesicle (GO:0061200)	5.936E-03	2.552E-02	208.09
GO Cellular Component	Clathrin-Sculpted Gamma-Aminobutyric Acid Transport Vesicle Membrane (GO:0061202)	4.243E-03	4.478E-02	208.09
GO Molecular Function	Melanocortin Receptor Activity (GO:0004977)	5.09E-03	4.478E-02	312.17
GO Molecular Function	Nitric-Oxide Synthase Binding (GO:0050998)	7.625E-03	4.478E-02	249.72
GO Molecular Function	O-methyltransferase Activity (GO:0008171)	7.625E-03	4.478E-02	156.05
GO Molecular Function	Benzodiazepine Receptor Activity (GO:0008503)	7.625E-03	4.478E-02	156.05
GO Molecular Function	RNA Polymerase II General Transcription Initiation Factor Binding (GO:0001091)	5.936E-03	2.552E-02	156.05

**Note**: GO refers to Gene Ontology; also, q-value and OR mean adjusted *p*-value and Odds Ratio, respectively.

**Table 4:** Diseases/drug assessments for candidate genes based on DisGeNET and GeDiPNet databases.

**Index**	**Name**	***P*-value**	**q-value**	**OR**
DisGeNET	Substance abuse problem	1.82E-22	1.98E-19	332.98
DisGeNET	Cocaine Dependence	2.13E-22	1.98E-19	328.35
DisGeNET	Heroin Dependence	7.30E-22	4.52E-19	547.55
DisGeNET	Addictive Behavior	6.87E-21	3.19E-18	242.29
DisGeNET	Mental disorders	1.14E-20	4.25E-18	202.27
DisGeNET	Impulsive character (finding)	1.51E-20	4.67E-18	623.34
DisGeNET	Abnormal behavior	5.76E-20	1.53E-17	177.65
DisGeNET	Anxiety Disorders	1.31E-19	3.04E-17	166.32
DisGeNET	Post-Traumatic Stress Disorder	3.14E-19	6.48E-17	284.04
DisGeNET	Nicotine Dependence	1.09E-18	2.02E-16	248.98
GeDiPNet	Mood Disorder	2.11E-11	6.11E-09	67.17
GeDiPNet	Schizophrenia	1.05E-10	1.22E-08	30.47
GeDiPNet	Mental Depression	1.28E-10	1.22E-08	31.62
GeDiPNet	Jacksonian Seizure	1.69E-10	1.22E-08	114.19
GeDiPNet	Clonic Seizures	4.13E-10	2.39E-08	97.65
GeDiPNet	Hypotonic Seizures	1.13E-09	5.42E-08	82.03
GeDiPNet	Minimal Brain Dysfunction	2.59E-09	1.07E-07	341.28
GeDiPNet	Status Marmoratus	3.13E-09	1.13E-07	323.3
GeDiPNet	Anxiety Disorder	1.61E-08	5.18E-07	36.01
GeDiPNet	Epilepsy	3.43E-08	9.91E-07	45.25

**Note**: q-value and OR sand for adjusted *p*-value and Odds Ratio.

**Table 5:** Results of metabolomics workbench for candidate genes.

**Index**	**Name**	***p*-value**	**q-value**	**OR**
1	Dopamine	1.018E-05	3.257E-04	665.97
2	12(13)-EpOME	5.090E-03	2.695E-02	249.72
3	3,4-Dihydroxyphenylglycol	5.09E-03	2.695E-02	249.72
4	9(10)-EpOME	5.09E-03	2.695E-02	249.72
5	Androsterone 3-Glucuronide	1.436E-02	2.695E-02	78.00
6	Testosterone Glucuronide	1.436E-02	2.695E-02	78.00
7	Morphine-3-glucuronide	1.436E-02	2.695E-02	78.00
8	Morphine-6-glucuronide	1.436E-02	2.695E-02	78.00
9	2-Methoxy-estradiol-17beta 3-Glucuronide	1.436E-02	2.695E-02	78.00
10	all-trans-Retinoyl-beta-glucuronide	1.436E-02	2.695E-02	78.00

**Note**: q-value represents the adjusted *p*-value and OR shows Odds ratio.

**Table 6:** Potential significant PGx annotations in relationships with cocaine, glucose/diabetes, and dopamine.

**Genes**	**Variant**	**Drugs**	**Association**	***P*-Value**	**Literature**
*DRD2*	rs2283265	Cocaine	Allele A is associated with increased risk of Death due to cocaine in people with Cocaine-Related Disorders as compared to allele C.	0.0008	PMCID: PMC3566726
*DRD2*	rs2283265	Cocaine	Allele A is associated with increased risk of Cocaine-Related Disorders due to cocaine as compared to allele C.	0.001	PMCID: PMC3055737
*DRD2*	rs1076560	Cocaine	Allele A is associated with increased risk of Cocaine-Related Disorders due to cocaine as compared to allele C.	0.003	PMCID: PMC3055737
*DRD2*	rs1125394	Cocaine	Allele C is associated with increased risk of Cocaine-Related Disorders due to cocaine as compared to allele T.	0.004	PMCID: PMC3055737
*DRD2*	rs1076560	Cocaine	Allele A is associated with increased risk of Death when exposed to cocaine in people with Cocaine-Related Disorders as compared to allele C.	0.014	PMCID: PMC3566726
*COMT*	rs4680	Glucose	Genotypes AA + AG is associated with increased concentrations of glucose in children as compared to genotype GG.	0.03	PMID: 25048416
*OPRM1*	rs1799971	Ethanol	Genotype AG is associated with increased response to ethanol in men as compared to genotype AA.	0.006	PMCID: PMC2925052
*OPRD1*	rs678849	Cocaine	Allele T is associated with decreased risk of Cocaine-Related Disorders due to cocaine as compared to allele C.	3.65E-07	PMCID: PMC3509227
*OPRD1*	rs678849	Disulfiram	Genotype CC is associated with increased response to disulfiram in people with Cocaine-Related Disorders as compared to genotypes CT + TT.	0.0001	PMCID: PMC8108700
*OPRM1*	rs3823010	Cocaine; Ethanol; Opioids	Allele A is associated with increased risk of Alcoholism, Cocaine-Related Disorders or Opioid-Related Disorders due to cocaine, ethanol or opioids as compared to allele G.	0.001	PMCID: PMC3164878
*OPRM1*	rs495491	Cocaine; ethanol; opioids	Allele G is associated with increased risk of Alcoholism, Cocaine-Related Disorders or Opioid-Related Disorders due to cocaine, ethanol or opioids as compared to allele A.	0.002	PMCID: PMC3164878
*OPRM1*	rs1799971	Cannabinoids; cocaine; ethanol; nicotine; opioids	Allele G is associated with decreased risk of Substance-Related Disorders due to cannabinoids, cocaine, ethanol, nicotine or opioids as compared to allele A.	0.00952	PMCID: PMC4752855
*OPRM1*	rs3778156	Cocaine; ethanol; opioids	Allele G is associated with increased risk of Alcoholism, Cocaine-Related Disorders or Opioid-Related Disorders due to cocaine, ethanol or opioids as compared to allele A.	0.01	PMCID: PMC3164878
*OPRM1*	rs62638690	Cocaine; heroin	Allele T is associated with decreased likelihood of Cocaine-Related Disorders or Heroin Dependence due to cocaine or heroin as compared to allele G.	0.02	PMCID: PMC3640707
*OPRM1*	rs1799971	3,4-methylene-dioxymethamphetamine; cocaine; ethanol; opioids	Genotype AG is associated with increased risk of Substance-Related Disorders due to 3,4-methylenedioxymethamphetamine, cocaine, ethanol or opioids as compared to genotype AA.	0.027	PMID: 31246565
*OPRM1*	rs2236256	Cocaine	Allele C is associated with decreased severity of Cocaine-Related Disorders due to cocaine as compared to allele A.	0.03	PMCID: PMC3425941
*OPRM1*	rs1799972	Cocaine; ethanol; nicotine	Genotype TT is associated with increased risk of Substance-Related Disorders due to cocaine, ethanol or nicotine in women as compared to genotypes CC + CT.	< 0.006	PMCID: PMC3117061
*SLC6A3*	rs28363170	Disulfiram	Genotype GGGGGCCCTGCATGCGTCCTGGGGTAGTACACGCTCCAGT/GGGGGCCCTGCATGCGTCCTGGGGTAGTACACGCTCCAGT is associated with increased response to disulfiram in people with Cocaine-Related Disorders as compared to genotypes GGGGGCCCTGCATGCGTCCTGGGGTAGTACACGCTCCAGT/del + del/del.	0.0001	PMCID: PMC6591035
*CHRNA5*	rs684513	Cocaine	Allele G is associated with increased likelihood of Cocaine-Related Disorders due to cocaine as compared to allele C.	0.01	PMCID: PMC3055642
*CNR1*	rs6454674	Cocaine	Genotypes GT + TT is associated with increased risk of Cocaine-Related Disorders when exposed to cocaine as compared to genotype GG.	0.004	PMCID: PMC3223560
*CNR1*	rs6454674	Cocaine	Genotypes GT + TT is associated with increased risk of Cocaine-Related Disorders when exposed to cocaine as compared to genotype GG.	0.017	PMCID: PMC3223560
*CNR1*	rs806368	Cocaine	Allele C is associated with decreased risk of Cocaine-Related Disorders when exposed to cocaine as compared to allele T.	0.017	PMCID: PMC3223560
*CYP2C19*	CYP2C19*17	Clozapine	CYP2C19 *17/*17 is associated with decreased likelihood of Diabetes Mellitus when treated with clozapine in people with schizoaffective disorder and Schizophrenia.	0.012	PMID: 28664816
*CYP2C19*	CYP2C19*1; CYP2C19*2; CYP2C19*3	Sulfonamides, urea derivatives	CYP2C19 *2/*2 + *2/*3 + *3/*3 (assigned as poor metabolizer phenotype) are associated with increased response to sulfonamides, urea derivatives in people with Diabetes Mellitus, Type 2 as compared to CYP2C19 *1/*1 (assigned as normal metabolizer phenotype).	0.039	PMCID: PMC9297921
*CYP2C19*	CYP2C19*1; CYP2C19*2; CYP2C19*3	Aspirin; Cilostazol; Clopidogrel	CYP2C19 *2/*3 is associated with increased maximal platelet aggregation when treated with aspirin, cilostazol and clopidogrel in high risk patients (acute myocaridal infarction, diabetes *e.g*.) as compared to CYP2C19 *1/*1.	< 0.007	PMCID: PMC3376439
*CYP2C19*	CYP2C19*1; CYP2C19*2; CYP2C19*3	Aspirin; cilostazol; clopidogrel	CYP2C19 *2/*3 is associated with increased high on-treatment platelet reactivity when treated with aspirin, cilostazol and clopidogrel in high risk patients (acute myocaridal infarction, diabetes *e.g*.) as compared to CYP2C19 *1/*1.	< 0.028	PMCID: PMC3376439
*DBH*	rs1611115	Levodopa	Genotypes CT + TT are associated with increased response to levodopa in people with Cocaine-Related Disorders as compared to genotype CC.	0.004	PMID: 24809448
*DBH*	rs1611115	Disulfiram	Genotype CC is associated with decreased severity of Cocaine-Related Disorders when treated with disulfiram in people with Cocaine-Related Disorders and Substance-Related Disorders as compared to genotypes CT + TT.	< 0.0002	PMCID: PMC3514624
*GABRA2*	rs11503014	Cocaine	Allele G is associated with increased severity of Cocaine-Related Disorders due to cocaine as compared to allele C.	0.001	PMCID: PMC3425941
*NOS1AP*	rs10494366	Glimepirid; glipizide; glyburide	Allele T is associated with increased risk of Hypoglycemia when treated with glibenclamide, glimepiride or glipizide in people with Diabetes Mellitus, Type 2 as compared to allele G.	0.003	PMCID: PMC8174577
*NOS1AP*	rs10494366	Repaglinide	Allele T is associated with increased response to repaglinide in people with Diabetes Mellitus, Type 2 as compared to allele G.	0.013	PMCID: PMC2860533
*SYT1*	rs2251214	Cocaine	Allele G is associated with increased risk of Cocaine-Related Disorders due to cocaine as compared to allele A.	0.0012	PMID: 31059723

## Data Availability

All data generated or analyzed during this study are included in this published article.

## LIST OF ABBREVIATIONS

CHRNA5: Cholinergic Receptor Nicotinic Alpha 5 Subunit
CNR1: Cannabinoid Receptor 1
COMT: Catechol-O-Methyltransferase
CSA: Cocaine Self-administration
CUD: Cocaine Use Disorder
CYP2C19: Cytochrome P450 Family 2 Subfamily C Member 19
DAT: Dopamine Transporter
D1-MSNs: Dopamine Receptor Type 1-expressing Medium Spiny Neurons
D2-MSNs: Dopamine Receptor Type 2-expressing Medium Spiny Neurons
DAT: Dopamine Transporter
DBH: Dopamine Beta-hydroxylase
DRD2: Dopamine Receptor D2
FDG-PET: Fluorodeoxyglucose Positron Emission Tomography
GABRA2: Gamma-aminobutyric Acid Type A Receptor Subunit Alpha2
GARS: Genetic Addiction Risk Score
GMIs: Gene-miRNA Interactions
GO: Gene Ontologies
NAc: Nucleus Accumbens
NOS1AP: Nitric Oxide Synthase 1 Adaptor Protein
OPRD1: Opioid Receptor Delta 1
OPRM1: Opioid Receptor Mu 1
OUD: Opioid Use Disorder
PGx: Pharmacogenomics
PPI: Protein-protein Interaction
PSU: Psychostimulant Use Disorder
RDS: Reward Deficiency Syndrome
SLC6A3: Solute Carrier Family 6 Member 3/Dopamine Transporter
SUD: Substance Use Disorder
SYT1: Synaptotagmin 1
